# The Psychosocial Effects of the COVID-19 Pandemic on Youth Living with HIV in Western Kenya

**DOI:** 10.1007/s10461-020-03005-x

**Published:** 2020-08-20

**Authors:** Jessica Dyer, Kate Wilson, Jacinta Badia, Kawango Agot, Jillian Neary, Irene Njuguna, James Kibugi, Elise Healy, Kristin Beima-Sofie, Grace John-Stewart, Pamela Kohler

**Affiliations:** 1 Department of Child, Family, Population Health Nursing, University of Washington, Box 359932, Seattle, WA 98104 USA; 2 Department of Global Health, University of Washington, Seattle, WA USA; 3 Impact Research and Development Organization, Kisumu, Kenya; 4 Department of Epidemiology, University of Washington, Seattle, WA USA; 5 Kenyatta National Hospital, Research and Programs, Nairobi, Kenya; 6 Department of Medicine, University of Washington, Seattle, WA USA; 7 Department of Pediatrics, University of Washington, Seattle, WA USA

**Keywords:** Adolescent, Kenya, HIV, Depression, Differentiated care

## Abstract

The objective of this study was to assess psychosocial effects of COVID-19 among adolescents living with HIV (ALHIV) in Kenya and to assess the feasibility of conducting behavioral surveys by phone. We adapted our protocol to administer telephone rather than in-person follow-up surveys and included questions about COVID-19. The majority of participants (99%) reported having heard of COVID-19; 23% reported no longer being able to go outside, 17% reported that they could no longer go to their regular clinic for medical care, and 3% reported that they could no longer get medication refills. PHQ-9 screening identified 9% (n = 45) with mild depression symptoms, and 1% (n = 3) with moderate-to-severe depression symptoms. Young adults 20–24 years old had more mild to severe depressive symptoms than the younger age groups (p < 0.001). Offering remote peer-support or mental health care, continuing to offer differentiated care services, and considering financial support will support the health and well-being of ALHIV.

## Introduction

Adolescents and young adults (ages 10–24) living with HIV (ALHIV) are a vulnerable group, with higher loss to follow-up, virologic failure, and mortality than older adults in HIV care [1]. COVID-19-related disruptions in social contact and health service delivery could negatively affect psychosocial and clinical outcomes, as well as reduce participation in research, among the approximately 190,000 ALHIV in Kenya [2]. Understanding the effects of the COVID-19 pandemic on ALHIV HIV care, as well as the feasibility of conducting remote research with ALHIV, can inform interventions to support this population during and after the pandemic.

After the first case of COVID was confirmed on March 13, 2020, the Kenyan government enacted policies to reduce the spread of COVID-19, including closing schools and higher-level learning institutions, limiting travel, banning social gatherings, enacting curfews, and requiring face masks in public spaces [3]. The Kenyan Ministry of Health (MOH) issued guidance for facilities providing HIV care that aimed to ensure continuation of antiretroviral treatment (ART) while decreasing clinic visit frequency. Recommendations included provision of up to 3 months of ART at each visit, regardless of age and viral load, even for ALHIV who typically get monthly refills. Additionally, facilities were encouraged to promote flexible ART delivery models, such as community-based ART groups. Prior to COVID-19, these differentiated treatment models were not recommended for adolescents below age 20 in Kenya [4].

These social and service delivery changes may specifically affect ALHIV, as they no longer have access to clinic-based adherence counseling and peer support groups or school-based support networks, increasing risk of isolation, anxiety, depression, and violence [5–7]. We assessed the psychosocial effects of COVID-19 within an ongoing cohort study of ALHIV.

## Methods

The Data-informed Stepped Care to Improve Adolescent Outcomes (DiSC) cohort includes 1386 ALHIV ages 10–24 years receiving HIV care at nine health facilities in Western Kenya. Following COVID-19-related clinical care changes in Kenya, we adapted our protocol to administer telephone rather than in-person follow-up surveys and included questions about COVID-19. All participants had previously provided informed consent at enrollment, and ethical review committees of Maseno University in Kenya and University of Washington, Seattle, United States, approved the phone interviews and additional COVID-19 questions.

During each call, study staff read a standard introduction, obtained oral consent to participate by phone, requested participants find a private location, and inquired about the battery life of the phone. If no private location was available or battery life was low, study staff would offer the option to re-schedule the survey. Special considerations were incorporated for 10–17 year old minors who had received parental consent to participate. Study staff first spoke with the caregiver on record and reminded them of the survey contents before speaking with the minor. To minimize risk of inadvertent disclosure of HIV status, study staff were provided information from enrollment data about participant knowledge of status and who else knows their status.

COVID-19 questions included whether ALHIV had heard of COVID-19 and how COVID-19 has affected their daily life (school has been closed, can no longer go to clinic, can no longer get medication refills, and/or can no longer go outside). Participants could also volunteer other ways COVID-19 has affected their lives; study staff paraphrased these responses and entered them into an open text field on an electronic tablet. Depression severity was assessed using the Patient Health Questionnaire (PHQ-9) tool, categorized as either none-minimal (score 0–4) or mild-severe (score ≥ 5). Psychological resilience was evaluated using the 2-item (score range from 0 to 8) Conner-Davidson Resilience Scale (CD-RISC), a measure of “bounce-back” and adaptability [8]. Categorical responses were compared across age groups using a Fisher’s exact test, and mean scores using one way ANOVA. Lastly, study staff could enter a comment about the phone survey experience. COVID-19 survey data collection began March 2020, and is ongoing.

## Results

### AYA Characteristics and Impact of COVID-19

The study team made 977 phone calls to 685 ALHIV participants and completed 486 surveys by phone. Thirty-one percent (n = 152) of ALHIV were 10–14 years old, 39% (n = 188) 15–19 years old, and 30% (n = 146) 20–24 years old; most of the older adolescents were female (84%) (Table 1). All of the younger adolescents reported being enrolled in school. Knowledge of one’s own HIV status was high among the two older groups (97%) and lower among 10–14 year olds (59%). Adherence was relatively high, with 6% of participants reporting missing their ARVs for 2 days or more in a row in the last 30 days.

**Table 1:** Impact of COVID-19, depressive symptoms, and resilience among ALHIV in Western Kenya by age group (N = 486)

	AYA 10–14	AYA 15–19	AYA 20–24	Overall	p-value
	N = 152	N = 188	N = 146	N = 486	
Characteristics					
Female	84 (55%)	108 (57%)	123 (84%)	315 (65%)	< 0.001
Enrolled in school	151 (100%)	169 (90%)	39 (27%)	359 (74%)	< 0.001
Knowledge of HIV status	90 (59%)	182 (97%)	142 (97%)	414 (85%)	< 0.001
Perceived mode of transmission^a^					0.006
Don't know	40 (44%)	49 (27%)	43 (30%)	132 (32%)	
Born with HIV	42 (47%)	104 (57%)	33 (23%)	179 (43%)	
Having sex	0 (0%)	10 (5%)	57 (40%)	67 (16%)	
Other/no answer	8 (9%)	19 (10%)	9 (6%)	36 (9%)	
Reported missing ARVs for 2 or more days in a row	3 (3%)	14 (8%)	9 (6%)	26 (6%)	0.63
COVID-19 impact					
Has heard of COVID-19	149 (99%)	184 (99%)	142 (97%)	475 (99%)	0.35
School has been closed (of those enrolled in school)	145 (96%)	157 (93%)	28 (72%)	330 (92%)	< 0.001
Can no longer go to the facility	27 (18%)	36 (19%)	22 (15%)	85 (17%)	0.62
Can no longer get medication refills	4 (3%)	4 (2%)	7 (5%)	15 (3%)	0.40
Can no longer go outside	28 (18%)	46 (24%)	39 (27%)	113 (23%)	0.20
Other	32 (21%)	62 (33%)	93 (64%)	187 (38%)	< 0.001
Reported > 1 COVID-related challenge	63 (41%)	93 (49%)	44 (30%)	200 (41%)	0.002
PHQ-9 depression severity^b^					
No or minimal (PHQ-9 score of 0–4)	140 (94%)	177 (95%)	114 (79%)	431 (90%)	
Mild-severe (PHQ-9 score of ≥ 5)	9 (6%)	9 (5%)	30 (21%)	48 (10%)	p < 0.001
Resilience^c^					
Mean CD-RISC (SD)	6.0 (± 1.6)	5.9 (± 1.4)	5.7 (± 1.8)	5.8 (± 1.6)	0.32

^a^Comparing “don’t know” with those that report a mode of transmission

^b^n = 479, 7 excluded for missing data

^c^n = 481, 5 excluded for missing data

Nearly all participants (99%) reported having heard of COVID-19, 23% reported no longer being able to go outside, 17% reported that they could no longer go to their regular clinic for medical care, and 3% reported that they could no longer get medication refills. There were no significant differences across age groups for these four ways in which COVID has affected participants. Among 359 ALHIV enrolled in school, 92% reported school closures. Of 486 adolescents, 41% (n = 200) reported more than one challenge related to COVID-19 with significant differences across age groups (p = 0.002).

PHQ-9 screening information was provided by 479 ALHIV (7 missing) and CD-RISC scores from 481 ALHIV (5 missing). Nine percent of ALHIV (n = 45) reported mild depression symptoms (PHQ-9 score of 5–9), and 1% (n = 3) moderate-to-severe depression symptoms (PHQ-9 score ≥ 10). Young adults 20–24 years old had more mild to severe depressive symptoms than the younger age groups (p < 0.001). Among 481 adolescents (5 missing), the mean CD-RISC score was 5.8 (± 1.6) (Table 1). No statistical differences were found in mean resilience scores across age groups.

### Other Responses: Social, Mobility, and Economic Effects

Over one third of participants (n = 187, 38%) selected ‘other’ and explained ways they have been affected with young adults 20–24 years old selecting ‘other’ the most frequently. Responses to this open-ended question were grouped into three categories: mobility and social effects, economic effects and other effects including disruptions to regular activities and feelings of fear (Table 2). Of the 486 ALHIV interviewed, 99 (20%) volunteered information about mobility and social impacts. Participants mentioned restricted opportunities to be with other people, including the inability to go to church, travel, attend markets, visit places, or leave their homes after a certain hour. Fourteen participants mentioned their social life was limited by the elimination of gatherings and inability to see friends. Ten participants reported people in their life dying from COVID-19.

**Table 2:** Open text responses describing ‘other’ ways COVID-19 has effected ALHIV in Western Kenya by age group (N = 187)

Category	AYA 10–14	AYA 15–19	AYA 20–24	Overall
	N = 32	N = 62	N = 93	N = 187
Mobility and social impacts				
Church	14 (44%)	21 (34%)	12 (13%)	47 (25%)
Mobility	2 (6%)	10 (16%)	12 (13%)	24 (13%)
Social life and greetings	4 (13%)	10 (16%)	4 (4%)	18 (10%)
Death and burial	6 (19%)	3 (5%)	1 (1%)	10 (5%)
Economic impacts				
Employment effects	1 (3%)	9 (15%)	40 (43%)	50 (27%)
Reduced income	0 (0%)	2 (3%)	6 (6%)	8 (4%)
Cost increase	1 (3%)	1 (2%)	9 (10%)	11 (6%)
Basic needs and food scarcity	0 (0%)	3 (5%)	5 (5%)	8 (4%)
Other economic	1 (3%)	1 (2%)	0 (0%)	2 (1%)

The most common economic effects reported (n = 50) were losing employment, not being able to access their job, or business being slow. The majority of those who mentioned employment issues were 20–24 years old (n = 40). One 22 year old participant noted that she can no longer sell in the market because it has been closed, which has led to *ruined* finances. Eleven participants described increases in general cost of living, food, and transportation; eight mentioned that they or their caregiver had reduced income due to COVID-19; and eight mentioned unmet basic needs, hunger, and food scarcity.

### Feasibility and Experiences Transitioning to Phone Surveys

Of 486 phone surveys conducted, study staff included a comment about the survey in 414 (85%). Overall, staff reported that the phone interviews went smoothly, with only 86 (18%) reporting challenges. The most frequently cited challenge was participant demeanor (n = 30, 6%). Study staff noted that “quiet” “shy” or “nervous” participants led to difficult surveys as a result of the participant not speaking a lot. Study staff also reported challenges with the telephone network (n = 28, 6%), and ‘interruptions’ (n = 21, 4%) including children crying, phone disconnections, rain, low phone battery, or the participant being in a public location. Participant comprehension was noted as a challenge in 7 surveys (1%), primarily among participants under 14 years old. In these instances, the study staff had to repeat and explain questions/answers to ensure comprehension, which lengthened survey duration.

## Discussion

These findings provide a glimpse into the effects of COVID-19 on ALHIV during the first 10-weeks following detection of the first case in Kenya. Despite school closure, social distancing, substantial economic impacts, and lost opportunities for social events, we found few ALHIV with moderate to severe depressive symptoms and resilience scores comparable to the normative population scores of students and young adults in other research [8]. Additionally, this study demonstrates that pivoting from in-person to surveys conducted by phone is feasible with ALHIV in rural Kenya.

It will be important to follow ALHIV over a longer period to understand sustained impact of social distancing and other COVID-19 effects such as adolescent mental health, resilience, and social support. In the meantime, proactive approaches to support ALHIV during COVID-19 are likely to be helpful. Peer-support groups or individualized mental-health care could be offered through telehealth or virtual platforms to individual reporting symptoms of anxiety or depression, or to compensate for missed clinic interactions during periods of social distancing. ALHIV under age 20 who maintain high levels of resilience without frequent clinic attendance may benefit from enrollment in differentiated care services, including support-person ART refills, multi-month prescriptions, and pharmacy-based refills, allowing those with the highest needs to attend and maintain safe distances during in-person care. Staggered scheduling of adolescent days at facilities to maintain social distance and decrease time in the clinic could further provide direct access to those most in need. Many ALHIV reported substantial economic difficulties as a result of COVID-19 social-distancing policies, and programs should consider financial subsidies or food vouchers to support the health and well-being of this population. Lessons learned from health services adaptations during COVID-19 offer promise to optimize ALHIV care both during the COVID-19 epidemic and beyond.
